# Measles vaccine coverage estimates in an outbreak three years after the nation-wide campaign in China: implications for measles elimination, 2013

**DOI:** 10.1186/s12879-015-0752-z

**Published:** 2015-01-22

**Authors:** Chao Ma, Fangjun Li, Xiang Zheng, Hong Zhang, Mengjuan Duan, Yanhua Yang, Lixin Hao, Qiru Su, Lance Rodewald, Bosong Guo, Shanliang Xiao, Huaqing Wang, Li Li, Junhua Li, Huiming Luo, Lidong Gao

**Affiliations:** National Immunization Programme, Chinese Center for Disease Control and Prevention, 27 Nanwei Road, Xicheng District, Beijing, China; Hunan Province Center for Disease Control and Prevention, Changsha, Hu Nan province China; Taizhou Prefecture Center for Disease Control and Prevention, Taizhou, Zhe Jiang province China; World Health Organization Office in China, Beijing, China; Longhui County Center for Disease Control and Prevention, Shaoyang, Hu Nan province China; Shaoyang Prefecture Center for Disease Control and Prevention, Shaoyang, Hu Nan province China

**Keywords:** Measles, Vaccination, Coverage, Estimation, Elimination, China

## Abstract

**Background:**

China is approaching measles elimination, but indigenous measles still circulates. County L in China has reported measles-containing vaccine (MCV) coverage rates >95% since 2000. Despite high reported coverage, a large measles outbreak occurred among young children in L County. We measured MCV coverage using 5 different methods during an investigation on this outbreak and compared our estimates with reported rates.

**Methods:**

Reported coverage rates are determined by aggregating clinic-based data across the county: doses administered in each clinic divided by the number of children registered in each clinic. Our methods estimated coverage for the 2010–2012 birth cohort, and were (1) administrative method: doses administered in clinics divided by the birth cohort recorded in the Statistical Year Book, (2) house-to-house convenience-sample survey of children living near cases, (3) vaccination clinic records review, (4) determination of a convenience sample of measles outbreak cases’ vaccination statuses and using the field vaccine efficacy outbreak equation to estimate population coverage, and (5) a seroprevalence survey using a convenience sample of residual blood samples from hospitals.

**Results:**

The measles outbreak totaled 215 cases, representing an incidence of 195.8 per million population. Our estimated MCV coverage rates were: (1) administrative method: 84.1%-87.0% for MCV1 and 80.3%-90.0% for MCV2, (2) in-house survey: 83.3% of 9–17 month children received MCV1, and 74.5% of 24–47 month children received MCV2, (3) clinic record review: 85.5% of 9–17 month children received MCV1, and 73.2% of 24–59 month children received MCV2, (4) field VE method: 83.6% of 9–47 month children received one or more MCV doses, and (5) serology: seropositive rates were <80% in the 12–17 and 18–23 month age cohorts.

**Conclusions:**

Compared with reported coverage >95%, our 5 coverage assessments all showed substantially lower coverage. China should evaluate guidelines for reporting vaccination coverage and identify feasible improvements to the assessment methods.

## Background

The World Health Organization Western Pacific Region has been striving to eliminate measles since 2005 [1] and set a goal to eliminate measles in the region by 2012. China adopted this goal and endorsed an action plan for measles elimination in 2006 that included continuing a two-dose measles-containing vaccine (MCV) strategy (administered at 8 months and 18–23 months of age), and called for routine measles vaccine coverage to be greater than 95% for both doses in every county, while using supplementary immunization activities (SIAs) to close immunity gaps among children [2].

Between 2004 and 2009, 27 of the 31 mainland provinces conducted unsynchronized province-wide measles SIAs targeting children aged 8 months through 14 years. In September 2010, China conducted a synchronized nationwide SIA targeting different age groups by province; children were vaccinated regardless of their prior vaccination status. Using the combined province-wide catch-up SIAs and nationwide follow-up SIA strategy, each province had covered their 1995–2009 birth cohorts through SIAs by 2010.

With these efforts, the measles incidence of China decreased dramatically, from 99.4 per million population in 2008 to 4.6 in 2012. However, indigenous measles virus outbreaks have been continuously reported, and a resurgence occurred in the end of 2012 that continued into 2013. Between January and October 2013, there were over 26,000 measles cases reported in China, which was 1.7 times more than case number of the same time period in 2011 and 4.6 times more than that in 2012. Of these cases, 68% were among children under 5 years of age [3], raising the concern that timely, 2-dose MCV coverage may not be high enough to eliminate measles outbreaks among young children.

In China, all vaccines are delivered through a public infrastructure with vaccination clinics and fixed-sites in urban and rural areas, and are offered to local children and to children new to the area who have registered for clinic services. Clinic immunization providers record each dose of vaccine administered, either through a vaccination card or a computerized immunization information system. Children who receive any routine vaccination in the clinics are registered in the clinic. Clinics are required to report vaccination coverage levels by dividing the number of children vaccinated in the clinics by the number of children registered in clinics. Reported coverage measured with this method has consistently been >98% in China [4,5], however, these rates may be too high because children not registered in a clinic are not included in the denominator. Survey-based coverage estimates in China have been lower than reported coverage. For example, a 2009 estimate showed coverage to be 91.1% for MCV1 and 84.3% for MCV2 [2]. A national coverage survey was conducted in 2011, using a stratified cluster sampling to select counties and Probability Proportional to Size sampling to select objects. Of the 4,681 children randomly sampled from 32 counties, 160 townships, and 480 villages, coverage rates for MCV1 and MCV2 were 99.4% and 93.35%, respectively [6].

In February 2013, a measles outbreak was reported in L County of Hu Nan province, with 215 measles cases, representing an incidence of 195.8 per million population. This county has reported administrative coverage of MCV greater than 95% since 2000. As a part of the province-wide catch up SIA in April 2009, L County targeted 8-month through 14-year-old children (birth cohorts of 1994 through part of 2008), and reported 98% coverage. As part of the 2010 nation-wide campaign, L County targeted 8-month through 4-year-old children (birth cohorts between October 2005 and 2009) and reported 98% coverage. Among all cases reported in the 2013 outbreak, 88% were among ≤3 year old children, and only 22% of 8–47 months old cases had received at least 1 MCV dose. The disease pattern raised our concern that there may be a discrepancy between reported and real MCV coverage rates.

The investigation of this outbreak provided an opportunity to determine whether coverage was as high as had been reported. We used 5 qualitatively different methods to estimate MCV coverage or protection levels in L County. We report the results of these measurements and contrast them to the reported coverage rates.

## Methods

Case-based measles surveillance data were obtained from the National Measles Surveillance System. A suspected measles case was defined using World Health Organization (WHO) criteria as a person with fever, rash, and one or more of the following symptoms: cough, coryza, or conjunctivitis. Key variables obtained included age, sex, address of residence, date of onset, occupation, MCV vaccination history, and measles-specific immunoglobulin (IgM) testing results. Case data were analyzed to describe person, place and time characteristics of the outbreak. During the field investigation, additional data were collected to estimate measles vaccine coverage and population susceptibility by using the following five methods.

### Administrative data coverage estimate

Routine immunization MCV1 and MCV2 coverage of the birth cohorts born after the 2010 measles SIA were estimated by using data from L County. The number of MCV1 and MCV2 doses administered through routine immunization in L County clinics in 2010, 2011 and 2012 were used as numerators, and the published data on number of new births reported in the Statistical Year Book for L County as denominators. Because this method is ecologic, the number of MCV2 doses can exceed the number of MCV1 doses when children change clinics. This method differs from the reported coverage method in the denominator – the reported coverage uses children registered in clinics, while this administrative data coverage estimate uses recorded births.

### House-to-house coverage survey

During the measles outbreak investigation, we identified a sample of children who had not acquired measles but who lived near a convenience sample of the measles cases. Investigators started from the house closest to the residence of each case, and visited additional houses one-by-one in a random direction to search for three healthy children of the same age as the case. Investigators reviewed vaccination certificate cards, and obtained records of each dose of MCV administered. If the certificate card was not available during in-house visit, investigators checked the immunization records in the vaccination clinic. In total, 190 parents of children aged 9 months to 15 years old were interviewed and their children’s vaccinations recorded.

### Immunization clinic records review

In the 9 townships of L County with the most measles cases reported, investigators reviewed clinic-registered children’s vaccination records. Records from a minimum of 40 children, 9–59 months old (birthdate between 1 January 2008 and 31 March 2012), were randomly selected from each township clinics’ list of registered children, and all MCV doses administered were recorded for each child.

### Coverage estimation by vaccine efficacy equation

In the 1980s, Orenstein and colleagues developed an equation to determine vaccine efficacy (VE) in the field during an outbreak [7]. The equation is PCV = (PPV − (PPV*VE))/(1 − (PPV*VE)), where PCV is the proportion of cases vaccinated; PPV is the proportion of the population vaccinated; and VE is vaccine efficacy. PPV (coverage) can be calculated if PCV is known and the vaccine has a known, relatively constant VE. We estimated MCV coverage of 9–47 months old children using the algebraic transformation of the field VE equation: PPV = PCV/(1 − VE(1 − PCV)) [7,8]. In this calculation, we assumed efficacy of a single-dose of measles vaccine to be 90%, and we assumed that the vaccination rate in case-patients with unknown vaccination history was the same as in case patients with known vaccination history [8].

### Prevalence of measles antibodies study

There are 2 major hospitals in L County, and in March 2013, we obtained residual serum specimens from these 2 hospitals’ clinical laboratories. Specimens were from non-fever, non-rash ill patients less than 35 years old. The specimens were shipped to Hu Nan Provincial CDC for measles-specific IgG testing to determine susceptibility to measles. The commercial Virion\Serion ELISA kit was employed to detect and quantify human IgG antibodies against measles virus in the sera. The laboratory results were interpreted according to the manufacturer’s instructions. Titers ≥1:200 were considered positive and titers <1:150 were considered negative. Sera samples for which the results were equivocal were retested using the same method; if again equivocal, we considered the result negative. Demographic information including age, gender, and location of residence were obtained, but individual-identifying data and immunization status and history of measles infection were not obtained. These data were analysed after determining sero-positivity and geometric mean titer.

### Ethical review considerations

Individual identifying information on the persons whose blood was tested for measles antibodies was not obtained by the investigators; the individuals were not contacted or interviewed; and informed consent was therefore not obtained. Obtaining and testing these samples of residual blood was approved by the Ethical Review Committee of Chinese Center for Disease Control and Prevention (Approval Notice No. 201410), recognizing that the right and the welfare of the subject are adequately protected; the potential risks are outweighed by potential benefits. Administrative (doses administered) data, coverage survey data, and vaccination record review data that are collected as part of a vaccine preventable disease outbreak investigation are considered by China CDC’s Ethical Review Committee to be exempt from IRB review. Informed consent is not obtained for accessing administrative, survey, and immunization clinic record data. Individual identifying data were not retained in analytic data sets.

## Results

### Outbreak description

**Figure 1:**
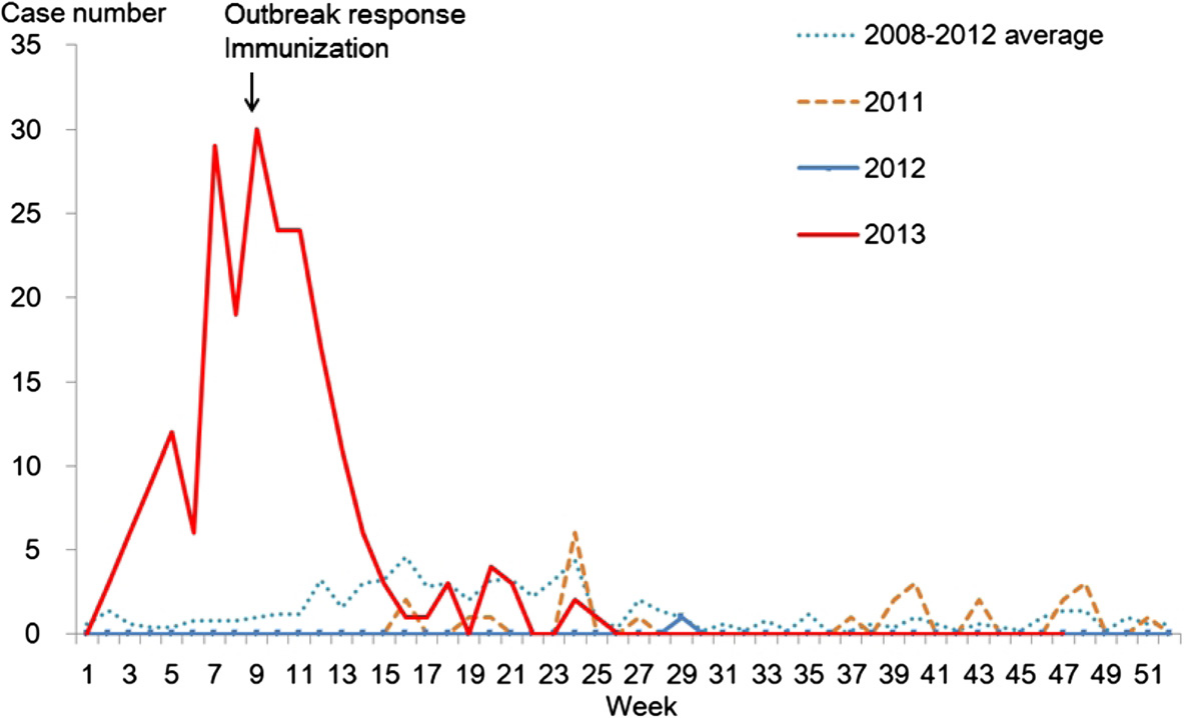
**Weekly distribution of reported measles cases, 2008-November 2013, L County, Hu Nan province, China.**

**Figure 2:**
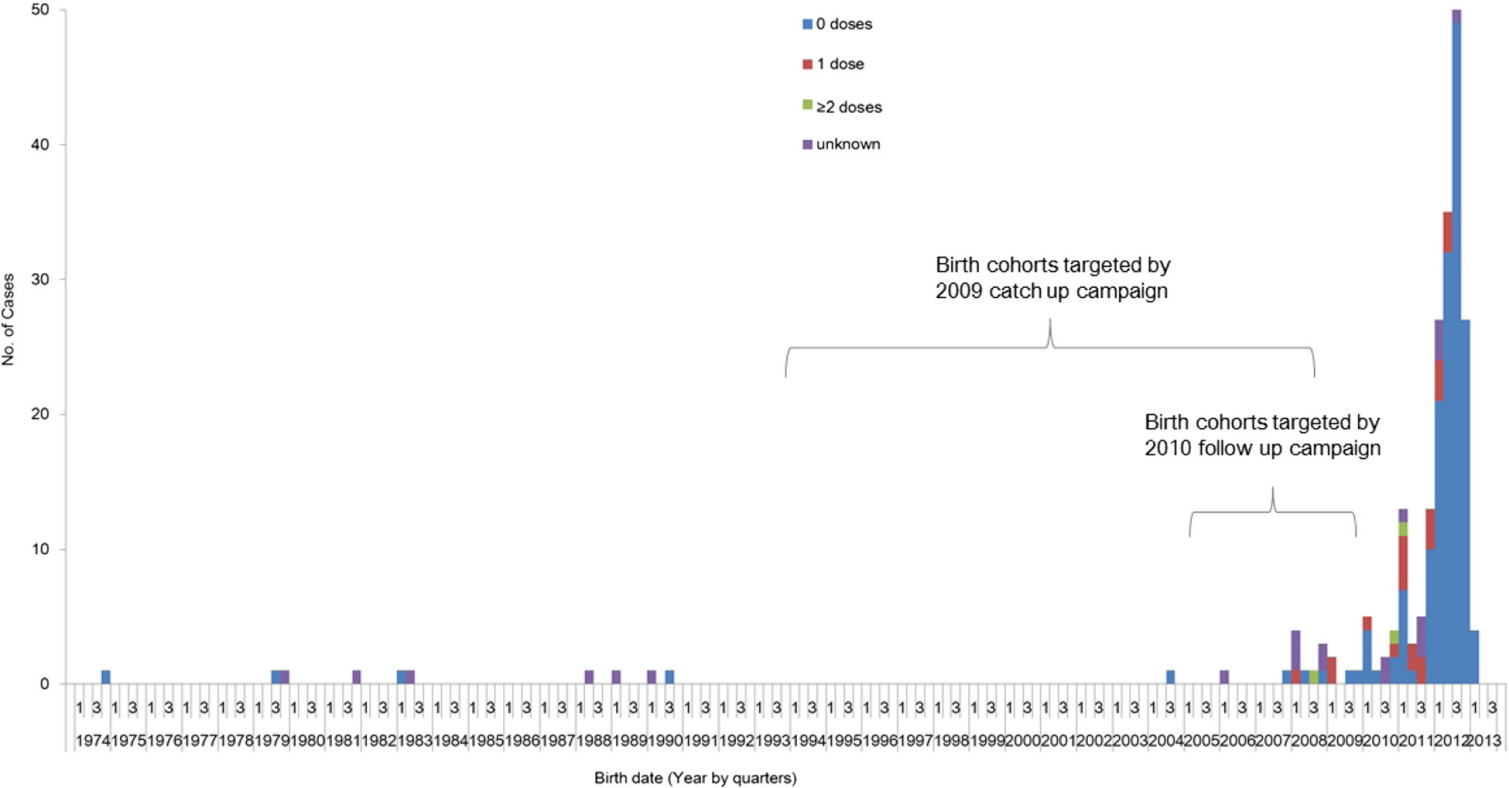
**Birth-date distribution of the 215 measles cases reported in L County, Hu Nan province.**

A total of 215 measles cases were identified in 24 of 26 townships in L County; 209 were laboratory confirmed and 6 were epidemiologically linked to a laboratory confirmed case. Of these, 153 cases (71.2%) were male, representing incidence (per million population) of 243.9 for male and 107.1 for female in this county. The outbreak started on January 9 and ended on June 19, 2013 (Figure 1). Of the 215 cases, 189 cases (87.9%) were born after 1 January 2010 and therefore had not been part of the 2010 nationwide measles campaign; 16 cases (7.4%) were born between 1 October 2005 and 31 December 2009 and were part of the 2010 nationwide campaign target age group; and 10 cases (4.6%) were >15 years old (Figure 2).

### Coverage estimation

From 2010 through 2012, the annual number of doses of MCV1 delivered through routine immunization in L County ranged between 13,718 and 14,452 doses, and the annual number of MCV2 doses ranged between 13,874 and 14,711 doses. Officially reported administrative coverage data for both MCV1 and MCV2 were >99% (Table 1).

**Table 1:** **Estimated MCV routine immunization coverage during 2010–2012, L County, Hu Nan province, China**

**Year**	**Dose**	**Reported coverage**			**Administrative estimated coverage**	
		**Number of vaccinated** ^**‡**^	**Number of target** ^**†**^	**Reported coverage (%)**	**Number of new birth cohort**	**Estimated coverage (%)**
2010	MCV1	14452	14526	99.5	16613	87.0
	MCV2	14657	14740	99.4	16725	87.6
2011	MCV1	13718	13728	99.9	16235	84.5
	MCV2	14711	14722	99.9	16354	90.0
2012	MCV1	14182	14193	99.9	16857	84.1
	MCV2	13874	13886	99.9	17267	80.3

^‡^The number of MCV2 doses can exceed the number of MCV1 doses when children change clinics.

^†^The “number of target” here, is the total number of children who have registered, and received routine immunization in vaccination clinics. It’s the denominator of administrative coverage.

The number new births in 2010 to 2012 reported in the Statistical Year Book ranged between 16,235 and 16,857. The calculated coverage estimate using the above doses administered data as the numerator and reported births as the denominator in 2010 through 2012 in L County ranged from 84.1% to 87.0% for MCV1, and from 80.3% to 90.0% for MCV2 (Table 1).

For the house-to-house survey, investigators interviewed 186 parents of children aged 9–179 months. Of 72 children aged 9–17 months who were age-eligible for MCV1, 83.3% (95% CI: 72.7-91.1) had received the first dose. Of 28 children between 18 and 23 months of age and eligible for MCV2, 85.7% (95% CI: 67.3-96.0) received MCV1, and 32.1% (95% CI: 15.9-52.4) received MCV2. Among 51 children 24–47 months old and due for MCV2, 98.0% (95% CI: 89.5-99.9) received MCV1, and 74.5% (95% CI: 60.4-85.7) received MCV2. All 35 children over 48 months old had received MCV2 (Table 2).

**Table 2:** **Coverage assessment from in-house interview, L County, Hu Nan province, China, 2013**

**Age group (months)**	**No. of children by MCV doses**				**Proportion of ≥1 doses (%, 95% CI)**	**Proportion of ≥2 doses (%, 95% CI)**
	**0 doses**	**1 dose**	**≥2 doses**	**Total**		
9-17	12	59	1	72	83.3 (72.7-91.1)	-
18-23	4	15	9	28	85.7 (67.3-96.0)	32.1 (15.9-52.4)
24-47	1	12	38	51	98.0 (89.5-99.9)	74.5 (60.4-85.7)
48-179	0	0	35	35	100	100
Total	17	86	83	186	90.9 (85.8-94.6)	-

In L County immunization clinics, 365 children’s immunization records were identified at random and were reviewed. Among 69 children aged 9–17 months, 85.5% (95% CI: 75.0-92.8) received MCV1. Among 46 children aged 18–23 months (eligible for MCV2), all received MCV1, and 41.3% (95% CI: 27.0-56.8) received MCV2. Among the 250 children aged 24–59 months, 73.2% (95% CI: 67.3-78.6) received MCV2 (Table 3).

**Table 3:** **MCV immunization status for the 365 registered children, L County, Hu Nan province, China, 2013**

**Age group (months)**	**Number of children (No., %)**				**Coverage (%, 95% CI)**	
	**0 doses**	**1 dose**	**2 doses**	**Total**	**≥1 dose**	**≥2 doses**
9-17	10	54	5	69	85.5 (75.0-92.8)	-
18-23	0	27	19	46	100.0 (92.3-)	41.3 (27.0-56.8)
24-59	2	65	183	250	99.2 (97.1-99.9)	73.2 (67.3-78.6)
Total	12	146	207	365	96.7 (94.3-98.3)	56.7 (51.5-61.9)

For the VE equation method, we obtained MCV vaccination status for 108 measles cases aged 9–47 months who were reported in the outbreak. Among these, 22 (20.4%) had received ≥1 dose MCV, while 86 (79.6%) had received no MCV doses. Using these rates to determine the proportion of cases vaccinated, and assuming 90% VE for one dose of measles vaccine, the vaccination coverage for this age group was estimated with the field VE equation to be 83.6%.

### Prevalence of measles antibodies

In total, 632 residual serum specimens were tested for measles IgG antibodies, and 74.5% (95% CI: 70.9-77.9) of these samples tested positive. Seropositive rates decreased from 60.0% (95% CI: 45.2-73.6) in 0–1 month group to 18.2% (95% CI: 5.2-40.3) in the 6–7 month age group. Seropositivity increased starting with the 8–9 months group and reached 90.1% (95% CI: 82.1-95.4) in the 24–47 month age group. Children 48 months to 15 years old had the highest seropositive rate, which was 95.8% (95% CI: 90.5-98.6). The 12–17 month and 18–23 month groups had seropositive rates less than 80%. GMT titers for each age group showed similar trends as the seropositive rate (Table 4).

**Table 4:** **Sero-prevalence of measles by age group, L County, Hu Nan province, China, 2013**

**Age group (months)**	**No. of tested**	**No. of positive**	**Positive rate (%, 95% CI)**	**GMT (IU/mL)**
0-1	50	30	60.0 (45.2-73.6)	163.05
2-3	37	15	40.5 (24.8-57.9)	163.23
4-5	34	10	29.4 (15.1-47.5)	43.27
6-7	22	4	18.2 (5.2-40.3)	27.07
8-9	20	5	25.0 (8.7-49.1)	34.25
10-11	27	10	37.0 (19.4-57.6)	38.9
12-23	71	54	76.1 (64.5-85.4)	360.16
24-47	91	82	90.1 (82.1-95.4)	822.43
48-179	120	115	95.8 (90.5-98.6)	914.99
180-419	160	146	91.3 (85.8-95.1)	743.84
Total	632	471	74.5 (70.9-77.9)	359.45

## Discussion

We have shown that reported MCV coverage, which is determined using clinic-registered children as the denominator, yields a higher coverage estimate than 5 qualitatively different coverage assessment methods that were obtained during a measles outbreak investigation. Because officially reported coverage estimates in China do use clinic-registered children as the denominator, reported coverage is likely to be higher than actual coverage. This higher-than-actual reported coverage may provide a partial explanation why indigenous circulation of measles has continued in China despite more than 25 years of a 2-dose MCV vaccination policy, the strategy of using measles SIAs, and high reported coverage with MCVs.

Conducting SIAs is useful to address coverage inequities and rapidly close population immunity gaps in targeted age groups. This has been demonstrated both in China and elsewhere in the world [9-12]. However, SIAs should not be considered superior to routine immunization [13], because they provide vaccination in an intermittent manner, allowing for accumulation of susceptible children between campaigns. Experience from previous elimination programs has demonstrated that a 2-dose measles vaccine policy has been highly successful in achieving and maintaining measles elimination status [14-16]. In the United States, there have been three efforts to eliminate measles targeting 1967, 1982, and 1996. Over the years, the U.S. experienced several failures, but systematically incorporated the lessons learned from each failure into subsequent efforts, and finally achieved the goal in 2000 [17,18]. Key lessons learned from the efforts include: (1) elimination requires very high MCV vaccination coverage by age 2 years, (2) a second dose of measles vaccine is needed to achieve satisfactory levels of immunity, and (3) coverage assessment is crucial [17,19].

In this outbreak, the 2010–2012 birth cohorts, which became age-eligible for measles vaccination following the 2010 campaign, had the highest incidence of measles. Fully 80% of the 108 MCV-eligible cases were unvaccinated. This pattern raised our interest in providing alternative estimates of true coverage as a check on the high reported coverage that was calculated using doses administered data with clinic-registered children as the denominator. Each result of the five independent measurement methods revealed that coverage of 2010, 2011, and 2012 birth cohorts was below 85%, which is much lower than the 95% target objective needed to eliminate measles, and much lower than coverage that was officially reported.

Several recently-published and unpublished studies also showed that low coverage of new birth cohorts after the 2010 campaign was a key factor contributing to China’s measles resurgence [8]. These outbreaks were due to failure to provide measles vaccine for children at recommended ages. Although the 2011 national survey showed high coverage for both MCV1 and MCV2 at the national level, low-coverage areas were also identified in the survey [5], indicating that “pocket areas” exist. A recently-published coverage survey also showed that coverage among a migrating population was lower than among local populations [20].

To eliminate measles in China, learning from other elimination programs and from domestic measles outbreaks is critically important. Several strategies are important for China. First, increasing and maintaining high (≥95%) coverage of both MCV1 and MCV2 through routine immunization is the top priority at this stage. The 1996–2009 birth cohorts (in some provinces back to the 1990 birth cohorts) have been targeted by SIAs, and many fewer cases have been reported from these age-group since 2011 [21]. The majority of new, potential susceptibles will come from new born children. Maintaining high coverage through routine immunization can minimize the number of susceptibles, and consequently avoid periodic epidemics. China is undergoing a large urban migration, leading to over 236 million migrant workers in 2012 [22], which complicates the administration of vaccines given in series and given during times of family movement. Conducting outreach to migrant children becomes an additional need for closing immunity gaps.

Second, delivering both MCV1 and MCV2 to 8-month and 18-month old children on time is essential. Several studies indicated that children born to vaccinated mothers lose their maternal antibodies earlier than children whose mothers were immune through infection [23-25]. Seroprevalence data from this study also showed the trend of rapidly decreasing seroprevalence - from 60% in 0–1 months to the lowest level of 18.2% in infants 6–7 months of age. China provides MCV1 at one of the youngest ages globally – 8 months. This young age is designed to protect children as early as possible, prior to risk of getting infection. To avoid future outbreaks, it is crucial to attain high coverage levels by timely vaccination [20], so that herd immunity can be robust enough to protect children too young to vaccinate.

Third, frequent monitoring of coverage is important to identify program areas that need to be strengthened. Outbreaks of measles can also be used to find program weaknesses, but outbreaks are lagging indicators. In contrast, coverage can be a leading indicator to find areas in need of additional effort before an outbreak occurs [17]. The unrealistically high coverage reported is not consistent with China’s current measles epidemiology. Having higher-than-actual reported coverage can undermine confidence in vaccination strategies by giving false sense that the programme cannot improve coverage, and may lead to a lack of understanding why outbreaks are occurring.

Fourth, coverage assessments should strive to include all children in an area. A purpose of coverage measurement is to identify areas at risk of disease outbreaks. It would not have been possible to use reported coverage to predict the L County outbreak because reported coverage ranged from 99.4% to 99.9% for each of the birth cohorts affected by the outbreak. Most likely, the restriction of the denominator to clinic-registered children resulted in missing children in the area who should have been registered in the clinic and vaccinated. Given the large urban migration in China, including all children in an area in coverage assessments will be an important component in an effective measles elimination strategy. When a measles outbreak occurs in a community, an initial step for public health officials can be to use population estimates to determine whether the number of children registered in community clinics is consistent with population estimates. If inconsistent, community based surveys using rigorous methodology may be indicated [26].

Fifth, enhancing outbreak analysis and response activities to close immunity gaps will be needed to eliminate measles. Given the imbalance of socioeconomic development and immunization program capacity, small area SIAs are likely to be needed as a supplement to routine immunization in less developed areas. These SIAs depend on the epidemiology of measles and identification of immunity gaps. Surveillance and outbreak analysis can provide additional information to determine the extent to which missed opportunities to vaccinate occur, and then decide which types of actions should be taken [27].

### Limitations

This study has several limitations, primarily the limitations of each of the 5 methods. The administrative method used Statistical Year Book birth records, and therefore would miss in- and out-migration among young children. The house-to-house survey is a convenience sample of neighborhood- and age-matched children, and is not representative of the entire county. Since these children did not get measles despite living near cases, their coverage may have been higher than the county average. The clinic review will miss children who never registered with the clinic, and can include children who moved away from the area. The VE equation method relies on the stability of the VE estimate. The serological survey was a convenience sample of children seen in the 2 hospitals, and previous studies comparing commercially available EIA assay versus the gold standard plaque reduction–neutralization assay have demonstrated that EIA was less sensitive, but is a reliable identifier of measles-seronegative individuals [28]. Although each of the 5 methods used in this study is imperfect and has advantages and disadvantages, all the 5 results come to the same conclusion that actual coverage is lower than reported coverage that is determined with a clinic-based denominator. We feel that a key strength of this study is the convergence of results from 5 qualitatively different methods. We therefore believe that our conclusion is supported by the evidence provided.

### Recommendation

We recommend review and evaluation of the methods for estimating officially-reported vaccination coverage levels in China, with a goal of identifying feasible coverage assessment methods that will provide useful information for the immunization program in China. Methods that include children not registered in immunization clinics should be sought.

## Conclusions

The L County measles outbreak was due to low routine immunization coverage, which was inconsistent with the high coverage rates that had been reported before the outbreak. Despite very high reported administrative coverage, measles epidemics will occur as a result of actual low vaccine coverage in socio-demographically clustered, mainly unvaccinated communities that accumulate susceptibles in new birth cohorts. Improving coverage assessments to identify areas of low coverage in time to prevent outbreaks will help the program achieve the goal of eliminating measles.

## Abbreviations

CDC: Center for Disease Control and Prevention
MCV: Measles containing vaccine
SIA: Supplementary immunization activities
VE: Vaccine efficacy
WHO: World Health Organization
